# Determination of Hazardous VOCs and Nicotine Released from Mainstream Smoke by the Combination of the SPME and GC-MS Methods

**DOI:** 10.1100/tsw.2010.127

**Published:** 2010-07-06

**Authors:** Sudhir Kumar Pandey, Ki-Hyun Kim

**Affiliations:** Atmospheric Environment Laboratory, Department of Environment and Energy, Sejong University, Seoul, Republic of Korea

**Keywords:** cigarette, smoke, solid phase microextraction, volatile organic compounds, nicotine

## Abstract

In this study, the contents of nicotine and volatile organic compounds (VOCs) in mainstream smoke (MSS) were analyzed using samples of four cigarette types consisting of two common brands (R and E) with full (F) and light (L) flavor, coded with R-F, R-L, E-F, and E-L. These cigarettes were also analyzed after removing the filter portions with the assignment of a new sample code of (N) as the third letter (e.g., R-L-N). A total of 44 VOCs (including nicotine) were quantified by the combination of the SPME and GC-MS methods. Out of the 44 VOCs, 10 were identified as hazardous air pollutants listed by the U.S. EPA, while their concentrations exceeded the reference exposure limits set by various agencies. A clear distinction was apparent in the concentration levels of VOCs between different brands or between full and light flavors. Nicotine concentrations varied greatly between different cigarettes types of the R brand, whereas such changes were insignificant in the counterpart E brand. This thus suggests that light-flavor cigarettes do not necessarily guarantee low doses of carcinogens (and tar) than regular cigarettes, as their differences can be balanced by the inhaling behavior of the smoker.

### INTRODUCTION

Tobacco smoke has been classified by the International Agency for Research on Cancer (IARC) as a known human carcinogen[1]. To the best of our knowledge, there is no threshold level of exposure to cigarette smoke to date. However, there is conclusive evidence to indicate that long-term smoking greatly increases the likelihood of developing numerous fatal conditions[2]. Nearly 4,800 chemical compounds have been identified as smoke components, including 60 known carcinogens[3]. However, as the analysis of smoke is hampered by its dynamic nature, only about 400 compounds have been subject to quantification. For this reason, there is a pressing need to analyze quantitatively all harmful components released via cigarette smoking in the form of environmental tobacco smoke (ETS).

Most analytical methods employed in ETS analysis have been developed on the basis of gas chromatography (GC) techniques with a special emphasis on some selective gaseous phase markers, such as nicotine and related nitrogen compounds, polycyclic aromatic hydrocarbons (PAHs), and a few volatile organic compounds (VOCs). The GC-flame ionization detection (FID) system has been successfully employed for the analysis of aromatic VOCs and total VOCs (TVOCs) contained in the ETS samples[4,5,6]. The use of the nitrogen-selective detectors (such as NPD) was also favored by some researchers for the quantification of nicotine or nitrogen-related compounds[5,7,8]. In recent years, mass spectrometry (MS) with GC has been helpful to determine selective markers such as nicotine, 3-ethyl pyridine, and 2-5 dihydrofuran[8,9,10,11].

In the present work, solid phase microextraction (SPME) combined with a GC-MS system was used to determine the dominant components existing in volatile fractions of mainstream smoke (MSS). The SPME method was selected for this study by considering its ease of operation, cost, and solvent-free nature. For this purpose, two different brands of cigarettes, each of which has both full- (regular) and light-flavor cigarettes, were selected and analyzed for their major volatile components released via MSS by focusing mainly on the toxic chemicals from the human health point of view. The classification of light and full flavor refers to the manufacturers' criteria based on tar and nicotine content that is relatively low for the light-flavor type than their full-flavor counterparts. Some ancillary experiments were also performed to observe, from different respects, the efficiency of filters in controlling the release of the harmful components. Moreover, the filter portion of each cigarette type was also evaluated for the possible release of any harmful volatile chemicals.

### MATERIALS AND METHODS

#### Collection of MSS

For the purpose of our study, four types of cigarette brands available in the Korean marketplace were selected with the original brand name (R and E) and by their tar and nicotine contents (light [L] vs. full [F] flavor), based on the classification of the manufacturers (e.g., R-L for light-flavor type of cigarette brand R). The tar and nicotine contents mentioned by the manufacturers were 4.50 and 0.50 mg (R-F), 1.00 and 0.10 mg (R-L), 0.50 and 0.05 mg (E-F), and 0.10 and 0.01 mg (E-L), respectively. The other details on these cigarette samples (i.e., cigarette length, filter length, diameter, and weight) are also presented in Table 1. These cigarette samples were also modified by detaching the filter portion with a sharp knife. Subsequently, these modified cigarette samples were assigned with the sample codes of R-L-N, R-F-N, E-L-N, and E-F-N. Here, the third letter N denotes "no" filter. In this way, four different types of raw cigarette samples (R-L, R-F, E-L, and E-F [with filter] and the four additional modified types (R-L-N, R-F-N, E-L-N, and E-F-N [without filter]) were analyzed for their smoke composition (Table 1). The cigarette sticks were burned at their lit end, and the smoke samples were collected in 10-L Tedlar bags (product code: 232-08, SKC, USA) connected to the mouth end of the cigarette through a vacuum sampler (ACEN, Korea), with the backup of air entering into the Tedlar bag. The flow rate of incoming air was maintained at a constant level (approximately 100 mL $min^{-1}$) by controlling the opening valve of the lung sampler in a clean room environment. As the samples were highly concentrated, they were further diluted (by 100 times in ultrapure nitrogen) in a 1-L Tedlar bag to fit in the MS calibration range. In addition to collection of MSS, the torn-apart filters from the sample codes of R-L-N, R-F-N, E-L-N, and E-F-N were also analyzed for any possible harmful chemical release. For this purpose, 1 L of ultrapure nitrogen was passed (at a flow rate of 100 mL $min^{-1}$ to match with the MSS sampling) through each detached filter portion and collected in Tedlar bags for final determination with the SPME-GC-MS method in a way similar to the MSS samples (Table 1).

**TABLE 1**
**Basic Information Regarding Different Cigarette Brands Analyzed for MSS Study**

| Order | Cigarette Brand | Flavor | Cigarette Length (mm) | Filter Length (mm) | Diameter (mm) | Weight (gm) | Type of Sample[a] | Abbreviation |
|---|---|---|---|---|---|---|---|---|
| 1 | R | Light (L) | 84 | 28 | 25.1 | 0.82 | With filter | R-L[b] |
| 2 | | | | | | | Without filter | R-L-N |
| 3 | R | Full (F) | | | | | With filter | R-F[b] |
| 4 | | | | | | | Without filter | R-F-N |
| 5 | E | Light (L) | 100 | 30 | 15.7 | 0.49 | With filter | E-L[b] |
| 6 | | | | | | | Without filter | E-L-N |
| 7 | E | Full (F) | | | | | With filter | E-F[b] |
| 8 | | | | | | | Without filter | E-F-N |

[a] MSS samples for each brand (or sample code) were collected in Tedlar bags connected through a lung sampler after burning three individual cigarette sticks consecutively for each sample type. All MSS samples were then collected on DVB-PDMS fiber by the SPME method. These samples were thermally desorbed in a GC injector and detected by a mass spectrometer.

[b] In addition to eight cigarette samples, the filter portions of each cigarette were also analyzed for possible VOC release after removing the filters from each respective brand and passing the pure $N_2$ through the torn-apart filters. These samples were also analyzed by the same analytical methodologies as those of the MSS samples.

### SPME Analysis

In order to induce the adsorption of gaseous components from MSS, SPME fiber (65 µm divinylbenzene/polydimethylsiloxane [DVB/PDMS], 57310-U [product number], Supelco, USA) was inserted into a 1-L Tedlar bag through its septum. Extraction of target compounds was performed at room temperature (25°C) for an exposure duration of 10 min. The desorption of smoke components was then accomplished by the insertion of an SPME fiber into the GC injector through a SPME injection sleeve (2-6375, Supelco) for a duration of 5 min. The injector system was operated at 230°C with a split ratio of 1:10.

### Chromatographic Separation and MS Conditions

The GC-MS system used in this study was a Shimadzu Gas Chromatograph 2010 QP plus (Shimadzu Scientific Instrument, Japan). For the analysis of smoke samples, our GC system was operated at the following temperature settings: (1) T (initial): 80°C (5 min), (2) T (ramping): 10°C $min^{-1}$ rate, and (3) T (final): 200°C (20 min). Chromatographic separation was achieved by Vocol column (60 m × 0.32 mm i.d. and 1.8-µm film thickness: Supelco) at a column flow rate of 1.2 mL $min^{-1}$ (99.999% pure He as carrier gas). The column flow rate was maintained in the linear velocity mode. Each running cycle was programmed to end at 37-min intervals.

As the detector was a quadrupole mass spectrometer, ion source of electron impact (EI) at 70 eV was used for ionization with ion source temperature = 200°C, interface temperature = 200°C, and detector threshold = 100. The total ion count (TIC) mode was applied to acquire the maximum number of compounds. For the TIC mode, a mass range of 35–250 m/z was selected to avoid the detection of the common air matrix.

### Quantification of VOCs

For the quantification of all the VOCs, a relationship was established for three VOCs (benzene [B], toluene [T], and xylene [X]) between the carbon ratio and the slope ratio based on a four-point calibration (10, 20, 40, and 200 ppb) of gaseous standard of BTX[12]. Such a relationship expressed in terms of the regression equation was used to estimate concentrations of other VOCs detected by our GC-MS system. To prepare the gaseous working standards (G-WS) of BTX, a primary standard (PS) gas was purchased at 20-ppm concentration (Ri Gas, Dae Jeon, Korea). For the preparation of G-WS, the PS was mixed proportionally with $N_2$ in a 1-L Tedlar bag. This mixing stage for G-WS was completed by a single-step dilution of the PS to the desired concentration with the aid of a gas-tight syringe. For instance, for the preparation of a 10-ppb concentration WS, 0.5 mL of PS gas was mixed with ultrapure nitrogen (999.5 mL) to make the final volume of 1 L. The detection limit (DL) values of VOCs were estimated in the sub-ppb range (e.g., in the range of 0.09 ppb [or 0.50 ng] for p-menthene to 0.44 ppb [or 0.74 ng] for acetonitrile) by considering the minimal peak area distinguishable from noise. The relative standard error (RSE) values were below the 5% level for a triplicate analysis of a 50-ppb gaseous standard mixture of benzene, toluene, and p-xylene.

## RESULTS AND DISCUSSION

### General Composition of MSS Across Different Brands

In Table 2, the results of our MSS analysis are summarized with respect to the concentration values of all the VOCs and nicotine. Although MSS may contain an enormous number of compounds, its quantification in this study was confined to a total of 44 compounds because of analytical feasibility (instrumental setups in this study: e.g., fiber characteristics of SPME) (Table 2). Nicotine was present in all the samples, as the most frequently quoted marker of ETS, regardless of brand or flavor type. Note that the environmental protection agency (EPA) of the U.S. has designated a total of 187 compounds in the list of hazardous air pollutants (HAPs)[13]. In order to classify the compounds present in cigarette smoke in this respect, the HAPs quantified in this study were evaluated in terms of their magnitude in concentration values (Table 2). As shown in Table 2, 10 compounds determined in this study belong to the HAP list. To evaluate the harmful nature of these air toxins, their concentrations were compared against various reference exposure limits (REL) (Fig. 1). As shown in Fig. 1, in the case of brand R (either light- [R-L] or full-flavor type [R-F]), many HAPs determined in this study (i.e., benzene, toluene, meta- and para-xylene, acetonitrile, methyl ethyl ketone, ethyl benzene, phenol, and styrene) exceeded the prescribed REL. On the other hand, in the case of brand E, toluene alone surpassed such criteria. In order to learn more about the relationship between odorant concentration and nuisance level, various agencies (such as the EPA and the Occupational Safety and Health Administration [OSHA] of the U.S.) have specified odor threshold values for numerous compounds. According to the odor threshold values for the above HAPs, some compounds, like benzene, ethyl benzene, and styrene, were found to exceed their threshold values in most of R rand sample types. However, brand E cigarettes exhibited such exceedance only in the case of styrene.

When the concentrations of MSS components were compared between two brands as a group (i.e., R and E) or between the four sub-brands (i.e., R-L vs. R-F, or E-L vs. E-F), their patterns were distinguished from a number of perspectives. For instance, the concentrations of many VOCs in cigarettes of full-flavor products (R-F or E-F) were higher by up to two times than those in light-flavor cigarettes (R-L or E-L). On the other hand, when this comparison is extended to the four sub-brands (i.e., R-L vs. E-L, or R-F vs. E-F), their concentrations in R brand were many times higher than their E brand counterparts.

### TABLE 2
## Concentration of Nicotine and VOCs Released in the Form of MSS from Different Brands of Cigarettes with Eight Different combinations

| Order | Compound | Concentration (ppb) | | | | | | | | Detection Limit (ppb) |
|---|---|---|---|---|---|---|---|---|---|---|
| | | E-L | E-L-N | E-F | E-F-N | R-L | R-L-N | R-F | R-F-N | |
| 1 | Benzene #[a] | 801 | 2,417 | 1,295 | 2,079 | 6,427 | 15,724 | 10,149 | 9,561 | 0.14 |
| 2 | Toluene # | 4,910 | 9,640 | 7,691 | 8,440 | 24,281 | 38,041 | 28,392 | 29,563 | 0.12 |
| 3 | m,p-Xylene # | 515 | 1872 | 1079 | 1693 | 2,286 | 3529 | 2423 | 5683 | 0.11 |
| 4 | Acetic acid | 1,127 | 3,143 | 1,776 | 2741 | 2,569 | 6,386 | 3,890 | 8,191 | 0.42 |
| 5 | Cyclopropane, ethylidene*[b] | 0.18 | 0.18 | 0.18 | 0.18 | 1,815 | 5,794 | 3,485 | 11,450 | 0.17 |
| 6 | Dimethyl ketone | 430 | 1,157 | 679 | 691 | 3,476 | 7,413 | 5,666 | 7,574 | 0.28 |
| 7 | Acetonitrile #* | 0.44 | 0.44 | 0.44 | 0.44 | 1,332 | 2,326 | 2,180 | 2,040 | 0.44 |
| 8 | 2,3-Butanedione | 524 | 860 | 568 | 673 | 2,976 | 4,464 | 4,059 | 3,459 | 0.21 |
| 9 | Methyl ethyl ketone # | 308 | 541 | 347 | 421 | 2,380 | 4,464 | 3,238 | 3,111 | 0.21 |
| 10 | Isovaleraldehyde | 121 | 144 | 113 | 281 | 1,013 | 1,534 | 1,322 | 1,030 | 0.21 |
| 11 | Methyl isopropyl ketone* | 0.18 | 0.18 | 0.18 | 0.18 | 2,779 | 6,469 | 4,285 | 7,075 | 0.17 |
| 12 | Hydroxyacetone* | 2,159 | 3,414 | 2,374 | 2809 | 0.29 | 0.29 | 0.29 | 0.29 | 0.29 |
| 13 | 2,3-Pentanedione | 244 | 488 | 394 | 407 | 1,826 | 2,004 | 1,900 | 1,950 | 0.17 |
| 14 | 2,5-Dimethylfuran | 459 | 909 | 766 | 805 | 2,572 | 4,859 | 3,610 | 4,715 | 0.14 |
| 15 | Propylene glycol* | 2,540 | 7,219 | 7,371 | 9514 | 0.22 | 0.22 | 0.22 | 0.22 | 0.22 |
| 16 | Methyl isopropenyl ketone* | 367 | 1,034 | 0.18 | 841 | 5,706 | 5,360 | 3,815 | 46,915 | 0.17 |
| 17 | Ethyl benzene # | 1,021 | 3,172 | 1,952 | 2795 | 3,375 | 5,587 | 3,622 | 13,680 | 0.11 |
| 18 | Cyclopentanone | 403 | 905 | 620 | 732 | 4,791 | 2,944 | 2,425 | 4,571 | 0.17 |
| 19 | 2-Methyl-pyridine | 555 | 1,051 | 665 | 858 | 992 | 851 | 762 | 3,252 | 0.14 |
| 20 | 2-Methyl pyrazine | 839 | 1,821 | 1,309 | 435 | 1,967 | 2,876 | 2,112 | 6,406 | 0.15 |
| 21 | Phenol # | 1,560 | 1,458 | 521 | 1513 | 5,825 | 9,358 | 6,185 | 13,708 | 0.14 |
| 22 | l-Limonene | 2,441 | 8,199 | 4,599 | 6913 | 7,128 | 8,292 | 5,932 | 40,072 | 0.08 |
| 23 | Acetoxyacetone* | 0.18 | 0.18 | 0.18 | 715 | 0.18 | 0.18 | 0.18 | 0.18 | 0.18 |
| 24 | 2-Cyclopentenone* | 1,512 | 3,219 | 0.18 | 2710 | 0.18 | 4,749 | 3,202 | 28,800 | 0.17 |
| 25 | Methyl isobutyl ketone #* | 0.15 | 484 | 0.15 | 0.15 | 0.15 | 0.15 | 0.15 | 906 | 0.15 |
| 26 | Isoamyl cyanide* | 0.15 | 455 | 244 | 0.15 | 0.15 | 0.15 | 0.15 | 1,728 | 0.15 |
| 27 | Nicotine | 7.20 | 6.96 | 5.34 | 5.03 | 6.90 | 13.2 | 15.7 | 43.7 | 0.09 |
| 28 | Styrene # | 479 | 1,001 | 748 | 884 | 1,480 | 2,935 | 2,842 | 5,531 | 0.11 |
| 29 | Ethanol* | 0.44 | 0.44 | 0.44 | 0.44 | 1,234 | 0.44 | 1,009 | 0.44 | 0.44 |
| 30 | Isobutyraldehyde* | 0.15 | 0.15 | 0.15 | 0.15 | 0.15 | 0.15 | 316 | 253 | 0.15 |
| 31 | 2-Pentyl alcohol* | 0.18 | 0.18 | 0.18 | 0.18 | 2,185 | 4,814 | 0.18 | 7,050 | 0.18 |
| 32 | 3-Methyl-pyridine* | 606 | 0.15 | 0.15 | 1130 | 0.15 | 0.15 | 0.15 | 2,396 | 0.15 |
| 33 | 2-Nonenal* | 0.10 | 0.10 | 0.10 | 0.10 | 0.10 | 0.10 | 0.10 | 1,093 | 0.10 |
| 34 | 2,6-Dimethyl-2,6-octadiene* | 0.09 | 0.09 | 0.09 | 0.09 | 0.09 | 0.09 | 0.09 | 2,753 | 0.09 |
| 35 | 2,3,6-Trimethyl-1,5-heptadiene* | 0.09 | 0.09 | 0.09 | 0.09 | 0.09 | 0.09 | 0.09 | 3,652 | 0.09 |
| 36 | 1,2,3-Trimethylbenzene* | 0.10 | 0.10 | 0.10 | 0.10 | 0.10 | 0.10 | 0.10 | 4,802 | 0.10 |
| 37 | p-Menth-1-ene* | 0.09 | 0.09 | 318 | 463 | 0.09 | 0.09 | 131 | 3,089 | 0.09 |
| 38 | Acetaldeyde #* | 0.44 | 0.44 | 0.44 | 0.44 | 0.44 | 1,091 | 0.44 | 0.44 | 0.44 |
| 39 | Butyl cyanide* | 0.15 | 320 | 0.15 | 0.15 | 0.15 | 0.15 | 793 | 0.15 | 0.15 |
| 40 | Isopentadiene | 206 | 440 | 288 | 330 | 0.15 | 0.15 | 0.15 | 0.15 | 0.15 |
| 41 | Methyl pyruvate* | 411 | 1,029 | 481 | 965 | 0.22 | 0.22 | 0.22 | 0.22 | 0.22 |
| 42 | 2-Methyl-2-cyclopentenone* | 1,564 | 0.15 | 0.15 | 0.15 | 0.15 | 0.15 | 0.15 | 0.15 | 0.15 |
| 43 | 3-Hydroxy-2-butanone* | 0.22 | 577 | 499 | 597 | 0.22 | 0.22 | 0.22 | 0.22 | 0.22 |
| 44 | 4-Methylpentanenitrile* | 0.15 | 0.15 | 0.15 | 393 | 0.15 | 0.15 | 0.15 | 0.15 | 0.15 |

[a] Compounds marked with "**#**" are listed in the HAP list of the U.S. EPA.

[b] For each compound marked with "*", the lowest concentration corresponds to its detection limit (DL) value.

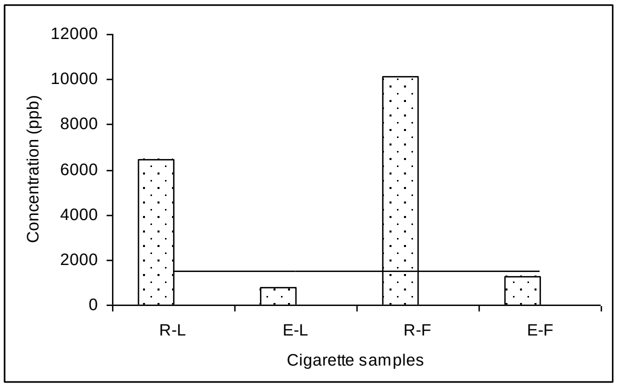

**(A) Benzene**

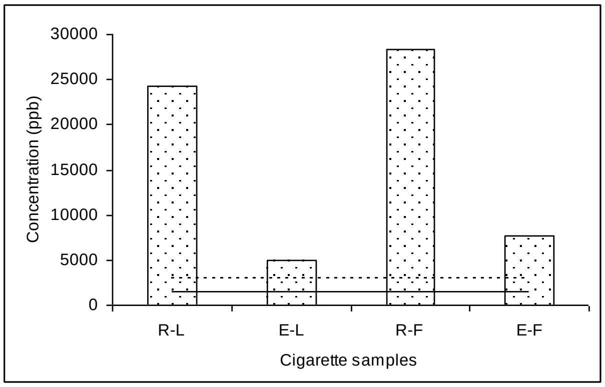

**(B) Toluene**

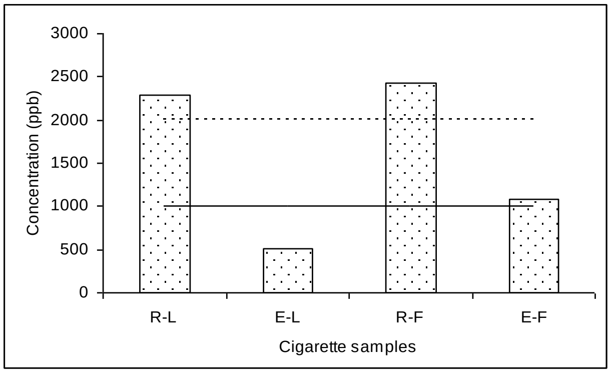

**(C) m,p-Xylene**

**(D) Acetonitrile**

**(E) Methyl ethyl ketone**

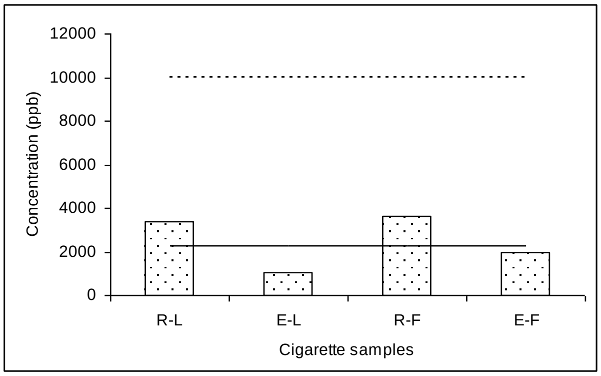

**(F) Ethyl benzene**

**FIGURE 1.** Comparison of concentration levels of HAPs (listed by the U.S. EPA) detected in different MSS samples with the exposure limits and odor threshold values prescribed by various agencies: (1) acute minimum risk levels (MRLs) by ATSDR for benzene, toluene, m-p-xylenes, acetonitrile, ethyl benzene, and styrene; (2) REL of NIOSH for acetaldehyde and phenol; and (3) reference concentration (RFc) of MEK by the U.S. EPA. Dotted lines show the exposure limits prescribed by various agencies and straight lines show the odor threshold values given by various agencies (i.e., benzene, toluene, methyl ethyl ketone, and styrene by the U.S. EPA and m-p-xylene by OSHA). *Continued next page.*

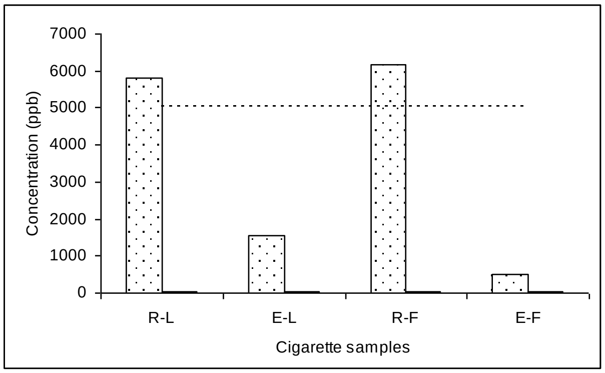

**(G) Phenol**

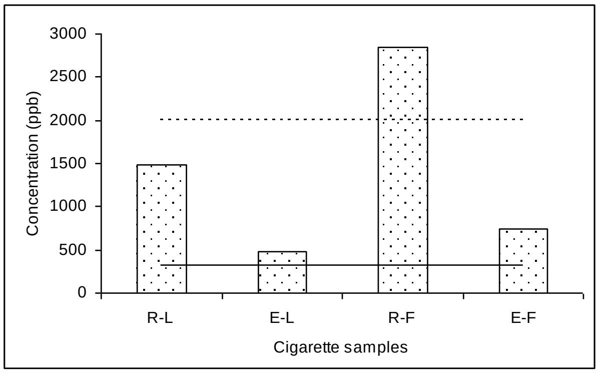

**(H) Styrene**

**FIGURE 1** continued.

Note that nicotine was detected in all the samples regardless of grouping scheme. However, it showed a distinctive pattern across different brands and between the sub-brands in terms of its concentration levels. In the case of R brand cigarettes, the results complied with the general expectation, such that relatively enhanced concentrations were seen dominantly in full-flavor (R-F) samples. Because the filters of light-flavor cigarettes have an abundance of tiny holes perforated to dilute the smoke with air, they may result in less inhalation of smoke contents (tar and nicotine). However, the smokers can compensate for this by puffing more deeply or by covering the perforated parts with their fingers (or lips). Hence, smokers of light-flavor cigarettes do not necessarily take in less doses of carcinogens and tar than smokers of full-flavor (or regular) cigarettes[14].

### Removal of Filters and Its Effect on Smoke Composition

A cigarette filter is used to reduce the amount of smoke, tar, and fine particles inhaled during the combustion of a cigarette. Filters can also reduce the harshness of smoke. In order to observe the efficiency of filters on MSS reduction, the smoke samples were also collected and analyzed after removing the filter portion of each individual cigarette type in the same manner to that of normal cigarettes (with filters). If the results are compared between with and without filters, the filterless data tend to experience a significant increase in the concentrations in many samples (Table 2). However, this increase was more significant in the case of light-flavor brands (Fig. 2). For instance, in the case of R-L samples, there was nearly two times an increase in the concentrations of almost all VOCs without the filters. The E-L samples also exhibited a similar trend, although not as strong.

Nicotine is often considered to be the most potent marker to characterize a particular cigarette brand because of its ubiquitousness in all cigarette products. When the nicotine concentrations measured in this study were evaluated between samples, a unique pattern was apparent among the two brands selected in this study (Table 2). The cigarettes with R brand showed an increase in nicotine concentration nearly two (for R-L) to three times (for R-F) after removal of the filter. However, to our surprise, the E brand cigarettes showed that their nicotine levels are not distinctively distinguished between all four sample types of E-L-, E-F, E-L-N, or E-F-N (Table 2). Although we cannot exclude the possible biases involved in sampling or analysis, this comparison suggests that nicotine content cannot simply be reflected by product type or flavor (Table 2).

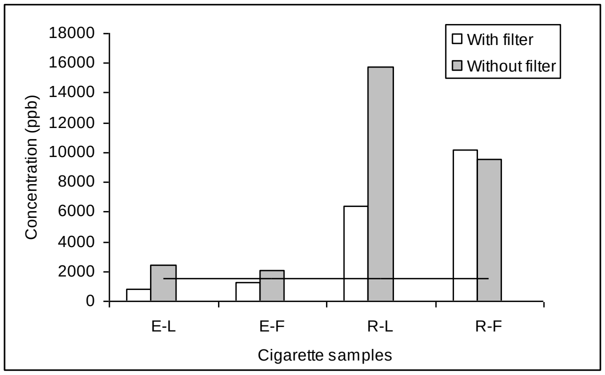

**(A) Benzene**

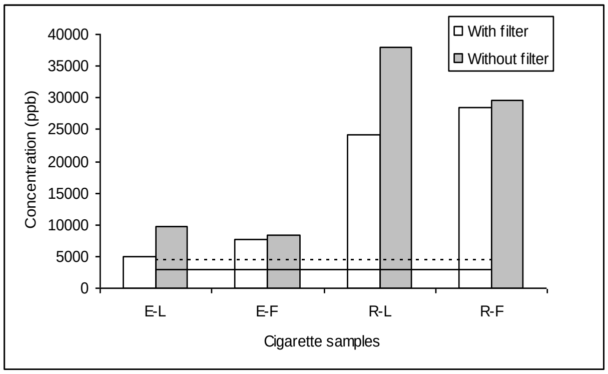

**(B) Toluene**

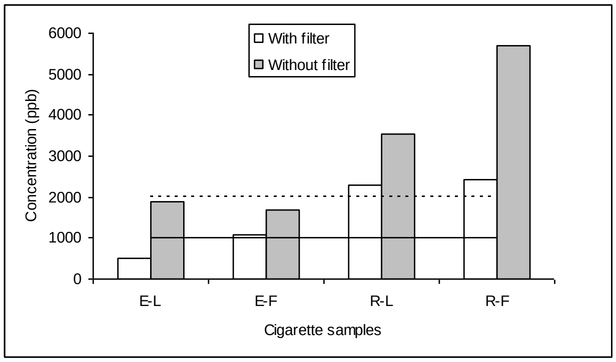

**(C) m,p-Xylene**

**(D) Acetonitrile**

**(E) Methyl ethyl ketone**

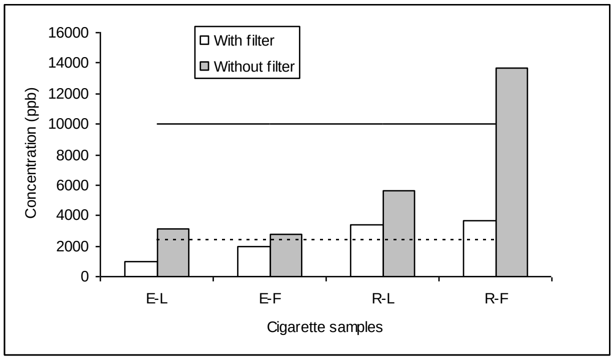

**(F) Ethyl benzene**

**FIGURE 2.** Comparison of concentrations of HAPs (listed by the U.S. EPA) from samples collected with filter vs. samples collected after removing the filter. (The comparison with REL and odor threshold values is shown with dotted and straight lines, respectively, as described in Fig. 1). *Continued next page.*

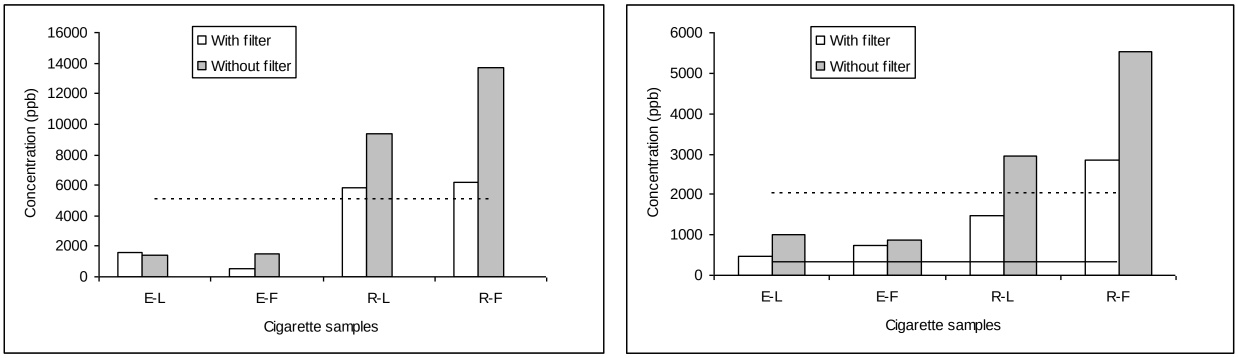

**(G) Phenol** **(H) Styrene**

**FIGURE 2** continued.

The classification of light- and full-flavor samples appears to be reflected mainly by such factors as the existence of perforations through which MSS can be diluted by inhaled air. Hence, if we compare emission concentrations of a cigarette for a particular brand between light- vs. full-flavor type, one may expect relatively good similarities between them to a certain extent. If the data sets obtained in this study are examined in that respect, the concentrations of the light type were not necessarily low, contrary to our anticipation. In fact, the concentrations of light-flavor cigarettes, if measured without filter, were comparatively higher in certain cases than their counterparts. For instance, in the case of E brand, the concentrations of most air toxins (benzene, toluene, xylene, methyl ethyl ketone, ethyl benzene, phenol, methyl isobutyl ketone, styrene, and nicotine) were comparatively high for the light flavor (E-L-N) than its counterpart (E-F-N) (Table 2). The pattern was similar in the case of R brand (R-L-N vs. R-F-N), when some compounds (benzene, toluene, acetonitrile, methyl ethyl ketone, and acetaldehyde) are considered. Hence, the observed patterns reconfirmed the fact that the selection of light cigarettes cannot assure the reduction in harmfulness to smokers, as there are many variables determining MSS compositions. If the holes of the filter are blocked partially or fully, one cannot exclude the possibility that the light-flavor cigarettes can also emit a similar quantity of harmful chemicals as their counterparts (regular ones).

### Analysis of Cigarette Filters

As we know that the filters of modern cigarettes are treated with many additives, some chemicals are also expected to come solely from the filter of the cigarette. To check for this possibility, the filters of each brand were separated to analyze any chemicals released as described earlier. As shown in Table 3, a total of seven compounds (including acetic acid, propyleneglycol, toluene, methylpyrazine, dimethylacetamide, phenol, and triacetin) were detected as the main components of the filter-only fraction across all the cigarette types; however, none was found to be harmful either by its concentration level or chemical nature.

### Remarks on the Sampling and Analytical Methods for Cigarette Smoke

Among many smoke-generation methods, one may consider both the U.S. Federal Trade Commission (FTC) and International Organization for Standardization (ISO) 4387 as the most common choices. These methods share the same criteria in terms of puff volume (35 $cm^3$), puff generation frequency (at every 60 sec), and puff duration (2 sec)[15]. Nonetheless, many researchers are still looking for some alternative techniques to quantify smoke constituents (e.g., Ye[16]). The method applied in this study is also explored to provide a simple and easy option to compare the relative composition of smoke across different brands or

**TABLE 3**
**Summary of Compounds Emitted from Filter Fractions of Different Cigarettes after Passing 1 L of Pure $N_2$ through Torn-Apart Filter**

| Order | Compound | Concentration (ppb) | | | |
|---|---|---|---|---|---|
| | | R-L | R-F | E-L | E-F |
| 1 | Acetic acid | 9.66 | 24.0 | 9.80 | 455 |
| 2 | Propylene glycol | 8.95 | 53.2 | 13.6 | 832 |
| 3 | Toluene*[a] | 7.05 | 0.12 | 0.12 | 0.12 |
| 4 | Methylpyrazine | 1.94 | 0.77 | 0.78 | 23.2 |
| 5 | Dimethylacetamide* | 1804 | 409 | 0.22 | 0.22 |
| 6 | Phenol* | 70.1 | 14.9 | 0.15 | 0.15 |
| 7 | Triacetin* | 33.2 | 0.10 | 25.8 | 989 |

[a] For each compound marked with “*”, the lowest concentration corresponds to its detection limit (DL) value.

treatments. Although the results derived by our approach can be different from those determined by the conventional smoking machines, our method was consistent enough to allow a comparison of the relative composition between different smoke components across different brands and treatments.

It has already been demonstrated that the optimization of the SPME method under suitable sampling and desorption conditions can yield fairly stable results (e.g., Pieraccini et al.[17]). However, SPME can suffer from a problem of the fiber saturation in ETS analysis. If such a problem occurs, it can eventually yield biased results for the components with very high concentration (e.g., nicotine)[16]. For this reason, a proper modification of the SPME technique is further desirable in terms of maintaining suitable sampling conditions for the selected fiber type[15]. Apart from the SPME method, the thermal desorption (TD) method that relies on the collection of smoke components on solid sorbents is also a good choice for smoke analysis as discussed in some of the recent studies[15]. However, there is a scarcity of sufficient data pertaining to basic criteria of that method, e.g., as breakthrough volume, desorption efficiency, and storage ability with respect to ETS in terms of the reliability or reproducibility.

### CONCLUDING REMARKS

In light of the damages resulting from cigarette smoke, a total of eight types of cigarette samples (consisting of light- and full-flavor products of the two major brands, with and without filters) have been examined with an application of SPME to GC-MS technique. The results were found to be good enough to quantify 44 VOCs (including nicotine) from cigarette smoke. Out of these 44 compounds, 10 were identified as HAPs listed by the U.S. EPA. Concentrations of these air toxins exceeded the REL prescribed by various agencies in many cases. Moreover, this study was able to distinguish between different brands and their sub-brands in terms of VOC concentrations and nicotine release. These differences can be ascribed to the difference in cigarette composition (i.e., differences in tar content and other additives) across different brands or between different treatment types (e.g., with or without filter) of the same brand. Although all the cigarettes were burned in a similar manner, the probable variability in the burning pattern of different cigarette samples (i.e., burning temperature) may also affect the concentrations of different MSS components across different sample types. In contrast to the MSS

samples, when filter portions were tested separately, no noticeable contributions were recognized with the absence of the harmful ETS components.

In summary, the level of VOCs and nicotine in smoke released by cigarettes varied noticeably across different brands and quality criteria (e.g., light and heavy). Based on the smoke composition of full vs. light flavor, or with filter vs. filterless cigarettes, it was evident that the selection of a light-flavor cigarette cannot assure less harm. This can be ascribed to many factors. For instance, the release of smoke components between light and heavy cigarettes can be highly variable with the behavior of a smoker. The study further suggests that there is a pressing need to evaluate the smoke compositions of cigarettes for their harmful contents with more diverse perspectives. At present, it is not easy to mimic the actual smoking behavior of smokers. However, efforts should be directed to attain the actual composition of smoke that smokers exhale along with the ETS that would otherwise be inhaled involuntarily by nonsmokers at the same time.

### ACKNOWLEDGMENTS

This work was supported by the National Research Foundation of Korea (NRF) grant funded by the Ministry of Education, Science and Technology (MEST) (No. 2009-0093848).
